# Association Between the ERVW-1 Gene Polymorphism (rs4727276) and the Pathogenesis of Preeclampsia: A Case-Control Study in Women From Northeast Brazil

**DOI:** 10.7759/cureus.106233

**Published:** 2026-03-31

**Authors:** Gelton Fonteles, José Juvenal Linhares, Anderson Weiny Barbalho Silva, Jackson do Nascimento Costa, Louhanna Pinheiro Rodrigues Teixeira, Lailton Oliveira da Silva, Aridenio Dayvid da Silva, Alisson Araujo Gomes, Thaís Torres Fonteles, Janssen Loiola Melo Vasconcelos

**Affiliations:** 1 Department of Health Sciences, Universidade Federal do Ceará, Sobral, BRA; 2 Department of Obstetrics and Gynecology, Universidade Federal do Ceará, Sobral, BRA; 3 Experimental Biology Center (NUBEX), University of Fortaleza, Fortaleza, BRA; 4 Department of Health Sciences, Centro Universitário Inta - UNINTA, Sobral, BRA

**Keywords:** clinical cases, dna, genotyping, hypertension, pregnancy

## Abstract

Background

Preeclampsia (PE) is a pregnancy-related hypertensive disorder and a major cause of maternal, fetal, and neonatal morbidity and mortality. Genetic susceptibility plays an important role in its pathogenesis, and molecular biomarkers involved in placental development, such as variants of the ERVW-1 gene, have gained increasing attention.

Methods

This study investigated the association between the ERVW-1 rs747276 polymorphism and PE in pregnant women from northern Ceará, Brazil. Clinical and genetic data were obtained from peripheral blood samples of women receiving prenatal care at the Ana Maternity Hospital of Santa Casa de Misericórdia de Sobral. The study included 64 women with PE and 76 normotensive pregnant controls. Genotyping was performed using RT-qPCR, and genotype and allele distributions were compared between groups.

Results

The CC, CG, and GG genotypes were identified and analyzed under dominant, recessive, and codominant inheritance models. A significant difference in genotype distribution was observed between the PE and control groups (p < 0.05). No association was found between the polymorphism and disease severity or time of onset. The findings suggest a possible association between the ERVW-1 rs747276 variant and susceptibility to PE.

Conclusions

The ERVW-1 rs747276 polymorphism may represent a potential molecular biomarker for PE; however, further studies are required to confirm its clinical applicability.

## Introduction

The World Health Organization estimates that approximately one quarter of maternal deaths in Latin America are associated with hypertensive complications during pregnancy [1]. Among these conditions, preeclampsia (PE) and eclampsia stand out as major contributors, although both are considered potentially preventable through adequate prenatal care. According to the Brazilian Federation of Gynecology and Obstetrics Associations, PE is a multifactorial and systemic disorder, clinically defined by the onset of hypertension and proteinuria after the 20th week of gestation in previously normotensive women [2].

Several studies have investigated the genetic factors involved in the pathogenesis of PE, with particular emphasis on the ERVW-1 gene, which encodes syncytin-1 [3-6]. This protein plays a crucial role in trophoblast differentiation and proper placental development [3,4]. Alterations in syncytin-1 expression have been associated with impaired placentation and increased severity of PE, suggesting its relevance as a genetic biomarker in the pathophysiology of this condition [5,4].

In addition to genetic determinants, Oliveira, Cunha, and Pissati reported that a positive family history and previous episodes of PE influence susceptibility to the disease [7-9]. Therefore, the identification of reliable biomarkers has become essential to facilitate early risk stratification and to support the development of targeted preventive and therapeutic strategies. Advances in this field may contribute to improved maternal and perinatal outcomes [10-12].

The ERVW-1 gene harbors several single-nucleotide polymorphisms (SNPs), including rs747276, rs18592540, rs56899772, and rs55516193 [4]. Among these, rs747276 has attracted attention due to its potential regulatory role in gene expression, possibly affecting syncytin-1 levels and, consequently, placental formation [3-6]. Preliminary findings from other populations suggest that variants in this region may be associated with altered transcriptional activity of ERVW-1. However, studies evaluating the association between the rs747276 polymorphism and PE remain scarce in Brazil, particularly in the Northeast region, where genetic background and socioeconomic factors may modulate disease expression.

Therefore, expanding research on the genetic mechanisms underlying PE is essential for the development of effective preventive and therapeutic approaches, especially in understudied populations. In this context, the present study aimed to investigate the potential association between the rs747276 polymorphism of the ERVW-1 gene and PE in pregnant women from northern Ceará, Northeastern Brazil.

## Materials and methods

Ethical considerations

The study was reviewed and approved by the Research Ethics Committee of Centro Universitário Inta - UNINTA, in accordance with the principles established by Resolution No. 466/12 of the Brazilian National Health Council. Ethical approval was granted under protocol number 6.055.778.

Study design and setting

This study was designed as a case-control study to investigate the association between the ERVW-1 gene polymorphism (rs4727276) and the occurrence of PE. This design was chosen because it allows comparison of genetic variants between affected individuals (cases) and normotensive pregnant women (controls), enabling the identification of potential associations between the polymorphism and disease presence in a defined population at a single time point. The study was conducted with pregnant women admitted to Ana Maternity Hospital of Santa Casa de Misericórdia de Sobral (SCMS), located in Northeastern Brazil. Laboratory procedures were performed at the Laboratory of Molecular and Developmental Biology (LBMD) and at the Experimental Biology Center (NUBEX) of the University of Fortaleza.

**Figure 1:**
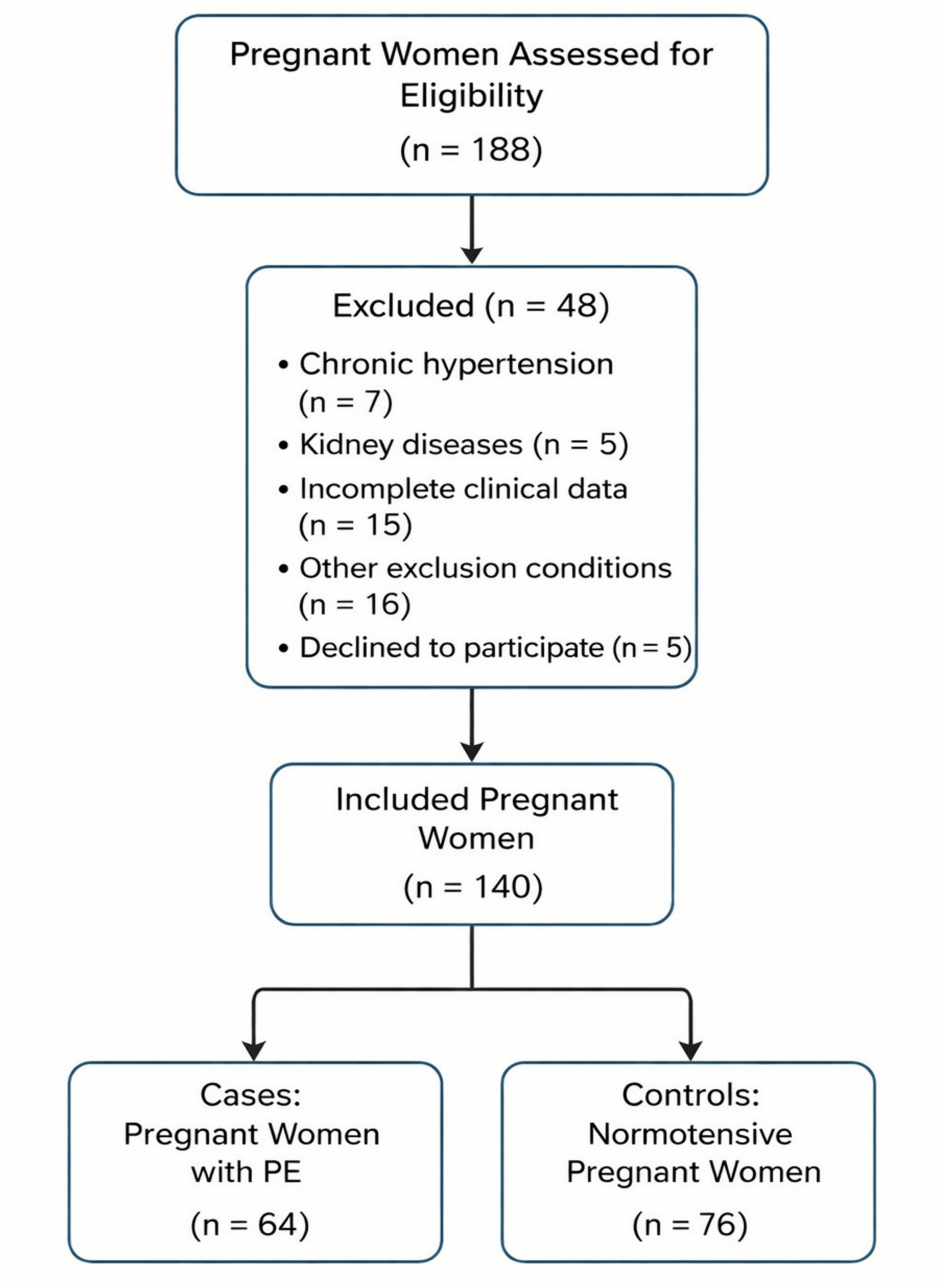
Flowchart of participant recruitment at the maternity hospital

A schematic representation of the study design is provided in Figure 1, and each methodological step is detailed in the following subsections.

Study population and sample

The study population consisted of pregnant women admitted to Ana Maternity Hospital of SCMS. Participants were allocated into two groups: women diagnosed with PE (case group) and normotensive pregnant women (control group).

Participants were prospectively recruited by convenience sampling between March and September 2023, based on maternity clinical records and face-to-face interviews. The case group included women diagnosed with PE according to the criteria established by the Brazilian Federation of Gynecology and Obstetrics Associations (FEBRASGO) [2], defined as (1) systolic blood pressure (SBP) ≥140 mmHg and/or diastolic blood pressure (DBP) ≥90 mmHg, confirmed by at least two measurements obtained at a minimum interval of four hours; and (2) proteinuria ≥300 mg in a 24-hour urine collection, or protein levels ≥1+ in two consecutive urine samples using dipstick testing, or ≥2+ in a single isolated urine sample, in accordance with the maternity clinical protocol.

Initially, 188 pregnant women were assessed for eligibility. Of these, 48 were excluded for not meeting the inclusion criteria or for declining to participate. Sample size calculation was performed considering a significance level of 5% and a statistical power of 80%, using SigmaPlot^®^ software (version 11.0; SYSTAT Software, Inc., San Jose, CA, USA), resulting in a minimum required sample of 65 participants per group. The flowchart presented in Figure 1 illustrates the screening process, exclusions, and final inclusion of participants according to the predefined eligibility criteria.

Inclusion and exclusion criteria

For the case group, eligibility criteria included pregnant women diagnosed with PE, aged over 18 years and with a gestational age greater than 20 weeks. Severe forms of PE were also considered eligible, including the presence of clinical signs or severe features such as headache, visual disturbances, scotomas, epigastric pain, nausea and/or vomiting, thrombocytopenia, or episodes of markedly elevated blood pressure (SBP >160 mmHg and/or DBP >110 mmHg), even in the absence of proteinuria.

The control group consisted of normotensive pregnant women who met the following inclusion criteria: age over 18 years, third trimester of pregnancy, no previous history of hypertension or PE, and SBP/DBP values ≤120/80 mmHg.

Exclusion criteria were applied equally to both groups and included multiple pregnancy; the presence of comorbidities such as coagulation disorders, cardiovascular disease, renal disease, autoimmune diseases, liver disease, or cancer; chronic or gestational hypertension; preexisting or gestational diabetes mellitus; isolated proteinuria; elevated liver transaminases in the absence of hypertension; chronic or inflammatory maternal diseases; intrahepatic cholestasis of pregnancy; hepatitis; and stillbirth.

Data collection

Data collection was performed at the bedside in the high-risk obstetrics unit of SCMS between March and September 2023. Participants received detailed information regarding the study objectives and procedures, without any interference in routine clinical care. Participation was confirmed through the signing of a written informed consent form.

Demographic and clinical data were obtained using a standardized data collection form. Variables included maternal age, gestational age, SBP and DBP, BMI, previous history of PE, and family history of PE in both groups. Laboratory parameters analyzed included hematocrit, hemoglobin, creatinine, urea, uric acid, leukocyte count, platelet count, aspartate aminotransferase, alanine aminotransferase, and lactate dehydrogenase (LDH).

All laboratory data were obtained from routine tests performed as part of high-risk pregnancy care. Quantification of 24-hour proteinuria was conducted using standard laboratory methods, while urine dipstick testing was used exclusively as a complementary screening tool.

After data collection and analysis, participants were classified into two categories, as described in Table 1.

**Table 1:** Classification of pregnant women according to the presence and severity of PE DBP, diastolic blood pressure; PE, preeclampsia; SBP, systolic blood pressure

Classification	Group	Description
Regarding the presence of PE, two groups were formed:	Case group	Pregnant women diagnosed with PE according to FEBRASGO criteria [2]
	Control group	Normotensive pregnant women without signs or symptoms suggestive of PE and with laboratory tests within normal limits, with no significant proteinuria
Regarding PE severity, the case group was subdivided into:	PE with severe features	Severe disease was characterized by the presence of at least one of the following findings: SBP ≥160 mmHg and/or DBP ≥110 mmHg, confirmed by two measurements obtained at a minimum interval of four hours; proteinuria ≥300 mg in a 24-hour urine collection or ≥2+ detected by urine dipstick testing; clinical manifestations such as persistent headache, visual impairment, scotomas, epigastric pain, nausea, and/or vomiting; and laboratory or fetal alterations, including elevated serum creatinine levels, increased liver transaminases, thrombocytopenia, fetal growth restriction, or evidence of placental insufficiency.
	PE without severe features (mild PE)	Non-severe disease was defined by SBP values between 140 and 159 mmHg and/or DBP values between 90 and 109 mmHg, accompanied by proteinuria of at least 300 mg in a 24-hour urine collection or a result of 1+ on two separate urine dipstick assessments, in the absence of any severe features.

PE was further categorized according to the timing of disease onset. Cases were classified as early-onset when clinical manifestations occurred before 34 weeks of gestation and as late-onset when onset was observed at or after 34 weeks of gestation [2].

For the genetic component of the study, buccal mucosa samples were collected using a cytological brush (cytobrush) for genomic DNA extraction. The collected samples were placed in properly identified tubes and stored at -80 °C until gene expression analysis was performed using RT-qPCR.

Laboratory assays

DNA Extraction

Genomic DNA was extracted using the PureLink™ Genomic DNA Mini Kit (Thermo Fisher Scientific, Waltham, MA, USA), following the manufacturer’s instructions. Briefly, 50 μL of saliva were mixed with 50 μL of lysis buffer and incubated at 95 °C for three minutes. After cooling, a stabilization buffer was added, followed by centrifugation. DNA concentration was measured using the Qubit^®^ dsDNA Assay Kit (Promega, Madison, WI, USA), and the extracted material was subsequently used for genotyping by RT-qPCR.

RT-qPCR

Genotyping of the rs4727276 polymorphism in the ERVW-1 gene was performed by RT-qPCR using TaqMan^®^ allelic discrimination assays. Reactions were prepared in 96-well plates with a final volume of 10 μL per well, containing Genotyping Master Mix, the specific probe for the rs4727276 SNP, genomic DNA, and ultrapure water. VIC™ and FAM™ fluorescent probes were used to detect the C and G alleles, respectively.

Amplification and allelic discrimination were carried out using a StepOne Plus™ real-time PCR system (Applied Biosystems, Waltham, MA, USA), including pre-read, amplification cycles, and post-read steps, allowing accurate determination of genotype distribution. Three genotypic variants were identified: homozygous CC, heterozygous CG, and homozygous GG. Genotypes were analyzed according to dominant, recessive, and codominant inheritance models.

Statistical analysis

Statistical analyses were performed using SigmaPlot^®^ software, version 11.0 (SYSTAT Software, Inc.). Data normality was assessed using the Shapiro-Wilk test. Continuous variables were expressed as mean ± standard error of the mean or as median, as appropriate. Comparisons between the PE and control groups for demographic variables, as well as biochemical and hematological parameters in women with PE, were performed using Student’s t-test for normally distributed data or the Mann-Whitney U test for non-normally distributed data.

Genotype distributions and allele frequencies were compared between groups using the chi-square test or Fisher’s exact test, when appropriate. Groups were further categorized according to the presence of each allele, either individually or in combination with the heterozygous genotype, to evaluate the influence of specific alleles on disease incidence and severity. Relative risk was estimated by calculating odds ratios through logistic regression analysis, with results presented alongside 95% CIs. A p-value < 0.05 was considered statistically significant.

## Results

A cohort of 140 women was included in the study, comprising 64 pregnant women with PE and 76 healthy pregnant controls, all of whom were genotyped for the rs4727276 polymorphism. Clinical parameters are summarized in Table 2. A statistically significant difference was observed between the PE and control groups with respect to BMI (p ≤ 0.000008). As expected, mean SBP and DBP values were significantly higher in women with PE (140.00 ± 20.70/83.33 ± 13.45) compared with those in the control group (113.00 ± 9.23/73.50 ± 8.75), with p-values of 0.00001 and 0.013, respectively. No significant differences were observed for the remaining variables.

**Table 2:** General characteristics of patients included in the study ^*^ p < 0.05 is considered significant (t-test) DBP, diastolic blood pressure; SBP, systolic blood pressure

Variables	Controls (n = 76)	Cases (n = 64)	p-Value	Statistics
Maternal age (years)	26.60 ± 5.87	27.94 ± 6.26	0.229	t = 1.210
Gestational age (weeks)	33.43 ± 3.50	33.13 ± 3.90	0.646	t = 0.461
BMI (kg/m²)	28.49 ± 5.11	33.04 ± 5.65	0.000008^*^	t = 0.4658
SBP (mmHg)	113.00 ± 9.23	140.00 ± 20.70	0.00001^*^	t = 5.020
DBP (mmHg)	73.50 ± 8.75	83.33 ± 13.45	0.013^*^	t = 2.619
Gestations (n)	1	1	0.424	X² = 3.870
Birth (n)	1	1	0.484	X² = 3.462

It is noteworthy that, although the mean blood pressure in the PE group is only slightly elevated, the standard deviation indicates considerable variability among participants. This reflects the inclusion of mild or early-stage PE cases, as well as patients who presented proteinuria or other severe features despite only moderate increases in blood pressure, according to the diagnostic criteria used. This individual variation explains the presence of some pregnant women with values close to those of the normotensive group.

Hematological and biochemical data for the PE group are summarized in Table 3 and compared between case and control groups. Significant differences were identified for lymphocyte counts and LDH levels when women with PE were compared with controls (p = 0.009 and p = 0.016, respectively). No statistically significant differences were observed for the remaining hematological and biochemical variables analyzed.

**Table 3:** Important hematological and biochemical parameters analyzed in the study ^*^ p < 0.05 is considered significant (t-test)

Variables	Controls (n = 76)	Cases (n = 64)	p-Value	Statistics
Hemoglobin (g/dL)	11.97 ± 1.43	11.60 ± 7.17	0.224	t = 1.223
Hematocrit (%)	35.70 ± 4.76	35.89 ± 4.71	0.852	t = 0.188
Leukocytes (×10⁹/L)	0.38 ± 0.96	0.88 ± 1.25	0.323	t = 1.015
Platelets (×10⁹/L)	223,878 ± 50,929	243,571 ± 100,283	0.269	t = 1.112
Lymphocytes (×10⁹/L)	12.83 ± 4.17	20.14 ± 6.64	0.009^*^	t = 1.283
Creatinine (mg/dL)	0.74 ± 0.11	0.81 ± 0.34	0.591	t = 0.541
Urea (mg/dL)	17.93 ± 6.23	20.36 ± 8.63	0.454	t = 0.755
Uric acid (mg/dL)	2.80 ± 1.56	3.96 ± 1.31	0.235	t = 1.208
Lactic dehydrogenase (U/L)	300.23 ± 151.72	573.63 ± 208.47	0.016^*^	t = 2.534

Genotype distributions and allele frequencies of the ERVW-1 rs4727276 polymorphism in the PE and control groups are presented in Table 4. A statistically significant difference in genotype distribution was identified between groups under combined genetic models (CC vs CG + GG and CG vs CC + GG; p = 0.011 and p = 0.05, respectively), suggesting an increased susceptibility to PE associated with these genotypes. Additionally, allele frequency analysis demonstrated a significant difference between groups, with the G allele showing higher prevalence compared with the C allele (p = 0.039).

**Table 4:** Distribution of genotypes and alleles of the rs4727276 polymorphism in the PE and control groups ^*^ p < 0.05 is considered significant (chi-square of Fisher’s exact test) PE, preeclampsia

Genotypes (rs4727276)	Controls (n = 76)	Cases (n = 64)	p-Value	OR (95% CI)	Statistics
CC	31(40.1%)	42(65.6%)	(CC vs CG + GG) p = 0.011^*^	2.77 (1.39-5.52)	X² = 8.588
CG	27(35.5%)	15(23.4%)	(CG vs CC + GG) p = 0.05^*^	1.14 (0.60-2.20)	X² = 2.418
GG	7(24.4%)	3(11.0%)	(GG vs CG + CC) p = 0.235	0.49 (0.12-1.96)	X² = 1.072
Alleles	Controls (n = 76)	Cases (n = 64)	p-Value		
C	58 (76.3%)	57 (89.0%)	(G vs C) p = 0.039^*^	2.52 (1.11-06.51)	X² = 3.848
Alleles					
G	34 (44.7%)	18 (28.1%)	(C vs G) p = 0.052^*^	0.48 (0.23-0.98)	X² = 4.106

The genotype distributions of the rs4727276 polymorphism in pregnant women with mild and severe or early and late PE are shown in Table 5. In terms of severity, no significant difference between the genotype distribution and degree of disease was observed (mild or severe). Furthermore, no significant difference was observed between the time of disease onset (early or late).

**Table 5:** Genotype distribution of the rs4727276 polymorphism in patients with mild and severe or early and late PE ^*^ p < 0.05 is considered significant (chi-square of Fisher’s exact test) PE, preeclampsia

Genotypes (rs4727276)	Mild PE (n = 10)	Severe PE (n = 4)	p-Value	OR (95% CI)	Statistics	Early PE (n = 33)	Late PE (n = 21)	OR (95% CI)	p-Value
CC	9 (90.0%)	2 (50.0%)	(CC vs CG + GG) p = 0.176	9.0 (0.52-155.4)	X² = 2.715	25 (75.7%)	14 (66.7%)	1.56 (0.47-5.23)	(CC vs CG + GG) p = 0.467
CG	1 (10.0%)	2 (50.0%)	(CG vs CC + GG) p = 0.176	0.11 (0.01-1.92)	X² = 2.715	7 (21.1%)	5 (23.8%)	0.86 (0.23-3.18)	(CG vs CC + GG) p = 1.000
GG	0 (0.0%)	0 (0.0%)	(GG vs CG + CC) p = 0.553	1	X² = 0.000	1 (3.2%)	2 (9.5%)	0.27 (0.02-3.50)	(GG vs CG + CC) p = 0.553

## Discussion

PE is a multifactorial syndrome whose pathophysiology involves a complex interaction between genetic, immunological, epigenetic, and environmental factors [2]. Although several studies have investigated polymorphisms associated with PE risk, few have specifically examined the role of the ERVW-1 gene, which encodes syncytin-1, a protein essential for trophoblastic fusion and proper placental development [13-15]. The scarcity of data on this molecular pathway limits the full understanding of the mechanisms involved, particularly in Latin American populations, which remain historically underrepresented in genetic research [16].

The ERVW-1 gene is highly sensitive to epigenetic modifications [17]. Studies have shown that hypermethylation of its promoter reduces syncytin-1 expression, contributing to impaired syncytiotrophoblast formation and, consequently, to placental dysfunction, a central mechanism in the pathogenesis of PE [18,19]. Reduced syncytin-1 expression has also been associated with placental hypoxia, increased inflammatory cytokines, and systemic endothelial injury [17].

These observations align with the results of the present investigation, which identified more pronounced hemodynamic impairment among women with PE, as evidenced by elevated SBP and DBP values [20]. Furthermore, the increase in blood pressure supports the association between placental ischemia and hypertensive disorders of pregnancy, underscoring the role of proper vascular function in maintaining normal gestational development [21].

Beyond hemodynamic factors, the inflammatory state observed in women with PE has been widely documented in the literature. In addition to classical risk factors such as elevated BMI, the present study identified significant differences in inflammatory markers, including lymphocyte count and LDH, between women with PE and normotensive controls [22,23]. These findings suggest that systemic inflammation may not only reflect disease severity but also actively contribute to its progression and related complications [21].

These data also support the hypothesis that systemic inflammation acts not only as a consequence of placental dysfunction but also as a modulator of disease evolution, as previously proposed by Redman and Sargent [24] and Brown et al. [25].

Genetic analysis revealed a significant association between the rs4727276 polymorphism of the ERVW-1 gene and PE, suggesting a hereditary predisposition to the condition. Allelic variations in regulatory regions of ERVW-1 may alter syncytin-1 expression and promote the establishment of a hostile placental environment [4,26]. The observed differences in genotype distribution and allele frequencies between the study groups highlight the complexity of the genetic factors involved in PE, whose clinical expression may be modulated by interactions with environmental factors [27].

Additionally, recent evidence has shown that broader placental epigenetic alterations also contribute to the pathophysiology of PE. For example, one study demonstrated that syncytin-1 expression is significantly reduced in placentas from women with PE, both at the mRNA and protein levels, accompanied by hypermethylation of the 5′-LTR promoter region of ERVW-1. This epigenetic pattern was associated with impaired trophoblastic fusion and deficiency of the syncytiotrophoblast layer, structures essential for proper placental formation [19].

The relevance of investigating genetically heterogeneous and admixed populations, including those from Northeast Brazil, is further supported by evidence showing that the prevalence of PE-associated variants varies considerably among individuals with African, Indigenous, and mixed ancestral backgrounds [28]. Characterizing risk variants in diverse populations enhances the understanding of the molecular pathways involved in the disease and may aid in the identification of biomarkers with broader applicability across different ethnic groups.

This study has important limitations. The total sample size of 140 pregnant women, 64 cases and 76 controls, although sufficient to detect general group differences, may be underpowered for stratified analyses (e.g., ancestry, severity of PE, early- vs late-onset disease), potentially explaining the absence of significant associations in these sub-analyses. Furthermore, no functional analyses were performed (such as syncytin-1 expression quantification or epigenetic assessment of ERVW-1 promoter methylation), which prevents confirmation of the biological effect of the rs4727276 variant in the context of PE. The cross-sectional design limits causal inference, and the absence of control for environmental factors, such as dietary habits, inflammatory exposures, nutrition, stress, or comorbidities, may confound the findings. Finally, the lack of longitudinal follow-up prevented the assessment of whether this polymorphism influences maternal or neonatal outcomes in the long term. Future studies should include larger samples, functional and epigenetic analyses, and ideally longitudinal designs to better elucidate the role of ERVW-1 in the pathogenesis of PE.

## Conclusions

The ERVW-1 rs4727276 polymorphism was found to be associated with PE in women from Northeastern Brazil, although no association was observed with disease severity or age at onset. These findings highlight the potential role of genetic variants involved in placental biology in the development of PE and support ongoing efforts to identify molecular markers for early risk assessment. However, the results should be interpreted with caution due to the modest sample size, particularly within a genetically heterogeneous population. Further studies with larger cohorts and functional analyses are needed to better elucidate the role of ERVW-1 in the pathophysiology of PE and its potential clinical implications.
